# Differential Selection for Translation Efficiency Shapes Translation Machineries in Bacterial Species

**DOI:** 10.3390/microorganisms12040768

**Published:** 2024-04-10

**Authors:** Heba Farookhi, Xuhua Xia

**Affiliations:** 1 Department of Biology, University of Ottawa, Ottawa, ON K1N 6N5, Canada; hfaro091@uottawa.ca; 2 Ottawa Institute of Systems Biology, University of Ottawa, Ottawa, ON K1H 8M5, Canada

**Keywords:** *Mycobacterium leprae*, *Mycobacterium tuberculosis*, translation efficiency, translation initiation, translation elongation, translation termination, RNA secondary structure, *rrn* operons, tRNA

## Abstract

Different bacterial species have dramatically different generation times, from 20–30 min in *Escherichia coli* to about two weeks in *Mycobacterium leprae*. The translation machinery in a cell needs to synthesize all proteins for a new cell in each generation. The three subprocesses of translation, i.e., initiation, elongation, and termination, are expected to be under stronger selection pressure to optimize in short-generation bacteria (SGB) such as *Vibrio natriegens* than in the long-generation *Mycobacterium leprae*. The initiation efficiency depends on the start codon decoded by the initiation tRNA, the optimal Shine–Dalgarno (SD) decoded by the anti-SD (aSD) sequence on small subunit rRNA, and the secondary structure that may embed the initiation signals and prevent them from being decoded. The elongation efficiency depends on the tRNA pool and codon usage. The termination efficiency in bacteria depends mainly on the nature of the stop codon and the nucleotide immediately downstream of the stop codon. By contrasting SGB with long-generation bacteria (LGB), we predict (1) SGB to have more ribosome RNA operons to produce ribosomes, and more tRNA genes for carrying amino acids to ribosomes, (2) SGB to have a higher percentage of genes using AUG as the start codon and UAA as the stop codon than LGB, (3) SGB to exhibit better codon and anticodon adaptation than LGB, and (4) SGB to have a weaker secondary structure near the translation initiation signals than LGB. These differences between SGB and LGB should be more pronounced in highly expressed genes than the rest of the genes. We present empirical evidence in support of these predictions.

## 1. Introduction

“The dream of a bacterium is to become two bacteria” [1], but the realization of this dream is often delayed by the rate of biosynthesis, especially the rate of translation [2,3,4,5,6,7,8,9], partly because most of the cellular dry weight is contributed by proteins. In well-studied enterobacteria, proteins account for more than half of the cell dry mass [10,11]. In *Escherichia coli*, the ratio of dry weight to wet weight is 0.2294 [12], and the ratio of protein weight to wet weight is about 0.2 [13]. Thus, proteins contribute about 87% (=0.2/0.2294) of the cellular dry biomass. It is not surprising that the growth rate of *E. coli* increases with the rate of protein production [11]. One, therefore, would expect a strong selection to optimize the translation machinery and mRNA features to increase translation efficiency.

### 1.1. Empirical Studies on Natural Selection for Optimizing Translation Efficiency

Efficient translation requires efficient initiation, elongation, and termination, as well as mRNA stability, and limits the rate of biosynthesis in both bacteria [3,6,7,14,15] and phages [5,16,17]. Translation initiation is often the limiting step [2,4,18,19,20]. Efficient initiation in bacteria generally requires (1) AUG as a start codon, (2) a well-positioned base-pairing between the Shine–Dalgarno (SD) sequence and the anti-SD (aSD) of the free 3′ end of the small ribosomal rRNA [21,22,23,24,25,26], and (3) no strong secondary structure that would embed the start codon or SD and consequently obscure it from being decoded by initiation tRNA or aSD, respectively [27,28,29]. 

With efficient translation initiation, translation elongation becomes rate-limiting [7,14]. Codon–anticodon adaptation is invariably observed in rapidly replicating organisms [2,6,30,31], especially in highly expressed genes [6,7,30,31,32,33,34,35,36]. The same pattern was also found in phages [5,9,16,17,37]. Experimental replacement of minor codons by major codons or vice versa typically leads to an increased or decreased translation rate in bacteria [7,38,39,40,41,42] and viruses [43,44]. Eukaryotic viruses such as HIV-1 tend to have the codon usage of their early and late genes adapted to their respective tRNA pools [45]. These results have led to the explicit formulation of codon–anticodon coevolution and adaptation theory, e.g., [6,46,47,48,49], and codon adaptation indices [7,50,51,52]. Although the relationship between codon usage optimization and protein production was briefly challenged [3], the relationship was fully reestablished by more detailed data analysis [7,14].

Translation termination in bacterial species is mediated by one or two release factors RF1 (decoding UAA and UAG) and RF2 (decoding UAA and UGA). All three stop codons can be misread by tRNAs in bacterial species [53,54]. The readthrough frequency is at least 10^−3^ to 10^−2^ for UGA in *Salmonella typhimurium* [55] and *E. coli* [56,57], and 1.1 × 10^−4^ to 7 × 10^−3^ for UAG, depending on the nature of the downstream nucleotides [54,58,59,60]. The readthrough rate is the lowest for UAA, at frequencies from 9 × 10^−4^ to less than 1 × 10^−5^ [54]. The readthrough error rate in the order of UGA > UAG > UAA has been consistently observed in multiple studies [57,61,62,63,64,65,66]. Empirical evidence supports the hypothesis that highly expressed genes prefer UAA stop codons [67]. 

Termination efficiency also depends on the nucleotide immediately downstream of the stop codon [68,69,70], leading to the proposal of the tetranucleotide stop signal including the +4 site [68,70,71,72,73,74,75,76]. Crosslinking was detected between RF2 and the +4 site in *E. coli* [77,78,79] and translation termination efficiency changes when different nucleotides were placed at the +4 site in *E. coli* [80]. In particular, the nucleotide usage bias at the +4 site is stronger in highly expressed genes than in low-expressed genes [70,81]. The best-documented case involves the translation of *prfB* mRNA (encoding RF2) in *E. coli* in which an inframe UGA stop codon is followed by nucleotide C [82,83,84]. When RF2 is abundant, the inframe UGA is decoded correctly to terminate translation, generating a short non-functional peptide. When RF2 is rare, the inframe UGA is not decoded. A +1 frameshift leads to the translation of GAC at a different coding frame, generating a functional RF2.

Almost all studies reviewed above focus on the optimization of mRNA but not on the translation machinery, which consists mainly of ribosomes, translation initiation factors, tRNAs, tRNA-charging enzymes, and release factors, as well as the energy that drives the translation machinery ([9,85] pp. 522–635). Given that ribosomes represent parallel translation machines, there are two lines of empirical evidence suggesting that short-generation bacteria (SGB) should produce more ribosomal proteins, rRNA [86,87], and tRNA [88] than LGB. First, the bacterial growth rate increases with the number of ribosomes in the cell [89,90,91,92]. Second, the number of ribosomes increases with rRNA abundance [93]. More ribosomes imply a greater need for tRNAs [94]. There should be more rRNA operons and tRNA genes in SGB than in LGB.

### 1.2. Two Approaches in Studying the Effect of Selection on Translation Optimization

The first approach to identifying the effect of selection on translation optimization is by within-species comparisons, especially by contrasting highly expressed and low-expressed genes (HEGs and LEGs) in rapidly replicating bacteria [5,8,9,33,49,67]. Because AUG and UAA represent stronger start and stop signals, respectively, than their alternatives, one would predict that HEGs should exhibit a stronger preference for AUG and UAA as start and stop codons than LEGs [5,8,9,67]. Because a strong secondary structure embeds translation signals such as a start codon, SD sequence, and stop codon, one would predict that HEGs should have a weaker secondary structure in sequence flanking these translation signals than LEGs [95]. Because early genes and late genes are translated with dramatically different tRNA pools, the two groups of genes should exhibit different codon optimization degrees [45]. The studies reviewed above constitute empirical tests of such predictions involving different groups of genes within individual species.

This paper focuses on differential selection for translation optimization among species. Relatively few studies are available, partly because of the difficulty in identifying differential selection. Occasionally, codon usage itself was taken as a proxy for selection [86], which is not a satisfactory approach. However, in at least four cases, such differential selection was identified with little controversy. The first case involves mitochondrial tRNA and protein-coding genes. Mitochondrial genomes of most multicellular eukaryotes encode only a single tRNA^Met/CAU^ (where Met is the amino acid methionine and CAU is the tRNA anticodon for a Watson–Crick base-pairing with the Met codon AUG) to decode both Met codons AUG and AUA (through wobble-pairing). In contrast, mitochondrial genomes in some bivalve species and tunicate species encode an additional tRNA^Met/UAU^ gene, which can decode AUA codons without wobble-pairing. One can predict that AUA codons should be used more frequently in mitochondrial protein-coding genes in those bivalve and tunicate species, which is true [31,96,97].

The second case of identifiable differential selection involves stop codons and release factors (RF1 decoding UAA and UAG, and RF2 decoding UAA and UGA) in bacteria [67]. If bacterial species X has more RF2 but less RF1 than species Y, then we would predict more UGA usage in species X than in species Y. This predicted pattern has also been documented in multiple bacterial species [67]. 

The third case involves phages differing in the presence of a lysogenic phase. Phage genomes in a lysogenic phase generally are not under selection for translation efficiency because the phage genome replicates by hitchhiking on the host genome. In contrast, phages without a lysogenic phase will be more commonly selected for translation optimization. This leads to the prediction that the former should exhibit stronger translation optimization than the latter, which is consistent with the empirical evidence [5,16].

The fourth case involves a key component of the translation machinery, i.e., a small subunit rRNA whose functions depend on stable secondary structures. Thermophiles are expected to have longer and more GC-rich stems than mesophiles, and this prediction is empirically supported [98]. The difference in growth temperature between thermophiles and mesophiles also affects the proportion of SD-led genes [99].

### 1.3. Hypothesized Impact of Differential Selection on Translation among Bacterial Species

Bacterial species differ dramatically in generation time. Under optimal culture conditions, the generation time is ~10 min in *Vibrio natriegens* [100,101], 16–20 min in *Vibrio cholerae* [102], 20–30 min in *Escherichia coli* [103], 30–70 min *Bacillus subtilis* [104], 103–107 min in *Haemophilus influenzae* [105], about 2 h in *Mycobacterium smegmatis* [106], 4–5 h in *M. abscessus* [107], 20–30 h in *M. tuberculosis* [15,108,109], and more than 7 days in *Mycobacterium leprae* [15,110]. Such dramatic differences in generation time have been dichotomized into r- and K-selection [111,112]. However, the evolutionary consequence of such differential selection has rarely been explored at the molecular level.

Translation machinery that needs to complete the task of protein synthesis in 10 min, as in the case of *V. natriegens*, should be under stronger selection for optimizing translation than that completing the task in 7 days, as in the case of *M. leprae*. The fitness of some parasitic bacteria is more related to survival against host attacks than to efficient translation. For example, *M. tuberculosis* cells build their private niches essentially isolated from the surrounding environment. This protective environment, while beneficial for the survival of the pathogen, also makes it difficult for the pathogen to gain nutrients and oxygen. If protein production is not the limiting factor in growth and reproduction, then one would not expect strong selection optimizing the translation machinery. Thus, we predict that selection for optimizing translation should leave much stronger signatures in rapidly replicating species such as *V. natriegens* than in slowly replicating species such as *M. tuberculosis* and *M. leprae*.

## 2. Materials and Methods

We include nine bacterial species with completely sequenced genomes and a well-documented generation time from the shortest to the longest among mesophiles under optimal growth conditions (Table 1). Generation time has typically been quantified only in model organisms (e.g., *E. coli* as a model species for Gram-negative bacteria and *Bacillus subtilis* as a model species for Gram-positive bacteria) and very harmful pathogens, so the sample of nine species is not representative of all bacteria. Also, the optimal growth condition from an experimenter’s perspective may not be the true optimal, so the generation time in Table 1 might be overestimated. However, the rank of the relative length of generation time (Table 1) should be correct, and the subsequent analysis will mainly be based on this rank. Our general prediction is that SGB should have more efficient protein-production systems than long-generation bacteria (LGB).

The optimal growth temperature for the first eight species in Table 1 is about 37 °C (or slightly lower). This is true not only for human pathogens and commensals but also for the free-living *B. subtilis* and *M. smegmatis.* The optimal growth temperature is about 30 °C for *M. leprae* when cultured with mouse foot pads [113] (as it has never been grown in vitro). *M. leprae* exhibits reduced growth above 33 °C presumably because of the lack of a heat shock response [106].

The RefSeq genomic sequences for the nine species were downloaded from GenBank by using the accession numbers in Table 1. The software DAMBE 7.3.0 [114] was used to extract coding sequences (CDSs), rRNA, and tRNA genes from the GenBank files. We classified the bacterial host genes into two expression groups: (1) known highly expressed protein-coding genes (HEGs) including small and large ribosomal protein genes, RNA polymerases, and some other genes known to be highly expressed (Supplemental Table S1), and (2) the rest of protein-coding genes not in the HEG group (REST). Pseudogenes were excluded from all analyses.

The RNA secondary structure could embed important translation signals such as the start codon, SD sequence, and stop codon and prevent them from being decoded by the translation machinery. We measured secondary structural stability based on the minimum folding energy (MFE) implemented in DAMBE [114], which uses the Vienna RNA fold library [115] for secondary structural characterization. A sliding window of 40 nt along the CDSs was used to characterize the change in MFE along the sequence. Codon adaptation was measured by the index of translation efficiency (*I_TE_*), which generalizes the conventional codon adaptation index (CAI) [50,51] to accommodate the background mutation rate [7].

The base-pairing between the SD sequence on mRNA and the aSD sequence on small-subunit (ssu) rRNA was hypothesized to position the start codon at the P-site to pair with the initiation tRNA [5,9,116]. The distance between the end of the ssu rRNA and the start codon (D_toStart_) is highly constrained in bacterial genes, especially highly expressed ones. A narrow distribution of D_toStart_ values suggests a stronger selection in terms of the SD/aSD pairing than a wide distribution. DAMBE [95] implements the calculation of D_toStart_.

## 3. Results

### 3.1. Differential Investment in Translation Machinery

Ribosomes represent parallel protein-production factories and their abundance in *E. coli* increases linearly with the growth rate [117,118]. Short-generation species may have not only more efficient factories but also more factories than LGB. Each ribosome features a set of 16S, 23S, and 5S rRNAs that are transcribed from the same operon and processed into individual rRNAs [119]. *E. coli* has seven rRNA operons (*rrnA* to *rrnE*, *rrnG*, *rrnH*) with promotors that are almost identical to the −10 and −35 consensus [120,121], suggesting a high demand for rRNA molecules met by both efficient and parallel transcription of multiple rRNA operons. The production of ribosomes in *E. coli* is limited by rRNA production [122], which explains why *E. coli* maintains multiple *rrn* operons in its genome for parallel transcription. A generalization of this would lead to the prediction that SGB should have more *rrn* operons than LGB. This prediction should also apply to tRNA genes because, with more ribosomes, more tRNA molecules are needed [123]. 

The two predictions are both supported by empirical evidence (Table 2), with the number of rRNA and tRNA genes decreasing highly significantly with an increasing generation time (*p* < 0.0001 for any rank-based nonparametric tests). The observation that SGB maintain more rRNA and tRNA genes in their genomes than LGB is consistent with the interpretation of stronger selection for translation efficiency in SGB than in LGB.

**Figure 1:**
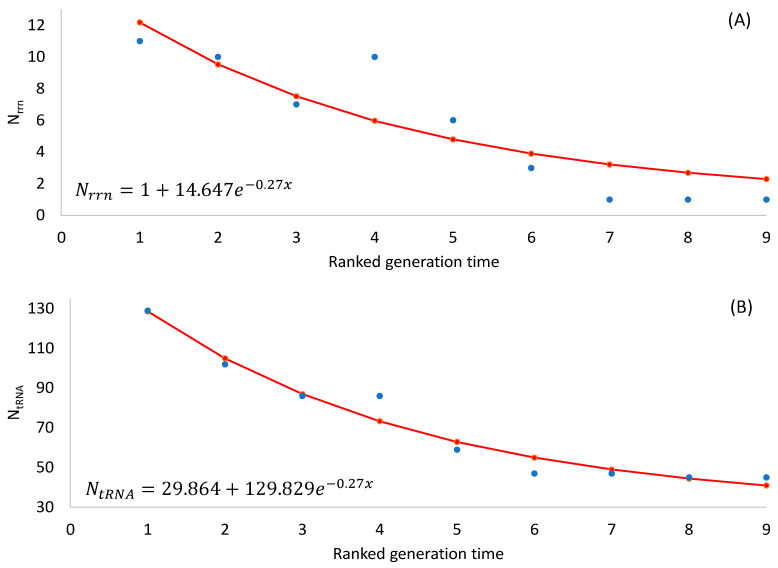
Fitted nonlinear equations to the observed data (blue dots). (**A**) Relationship between N_rrn_ and RankGT in Table 2. (**B**) Relationship between N_tRNA_ and RankGT in Table 2.

Note that an organism needs at least one *rrn* operon for translation. Ribosomal RNAs form the core of ribosomes with all important sites such as A, P, and E sites, with ribosomal proteins padding the surface of a ribosome [124]. Thus, the number of *rrn* operons cannot be less than 1. Also, there should be a minimum set of tRNA genes to decode all 61 sense codons. One might use the following two equations to model the numbers of *rrn* operons and tRNA genes, respectively:

(1) $$N_{rrn}=1+ae^{-bx}$$

(2) $$N_{tRNA}=c+ae^{-bx}$$

where *x* is RankGT in Table 2. Equation (1) ensures a minimum *N_rrn_* of 1. Equation (2) ensures a minimum *N_tRNA_* of c, which is estimated by the least-squares approach to be 30 (Figure 1B). The minimum number of tRNA genes required for decoding all sense codons in a natural translation system is observed in a vertebrate mitochondrial genome that encodes 22 tRNA genes. 

### 3.2. Differential Preference for Start Codon AUG

Among the three canonical start codons (AUG, GUG, and UUG), the translation initiation efficiency is consistently in the order of AUG > GUG > UUG [125,126]. HEGs in bacteria and bacteriophages tend to prefer AUG as a start codon [5,9,18,125,126,127]. This is true for all nine bacterial species in which the percentage of AUG (AUG%, Table 3) is consistently higher in HEGs than those in the REST (Table 3), which includes all genes not in the HEG group and consequently contains both highly expressed and low-expressed genes. This suggests that AUG is the most efficient start codon, as is consistent with previous empirical studies based on within-species comparisons [5,8,9].

Given that AUG is the most efficient start codon, one would predict that SGB should use more AUG codons as start codons than LGB. The AUG% in Table 3 is indeed strongly associated with the ranked generation time in Table 1, as predicted. However, the AUG% is also affected by the genomic GC% because GC-rich genomes tend to have more GC-rich codons [31]. This is best illustrated by focusing on start codons AUG and GUG. AT-rich genomes tend to have AT-biased mutations, which favor AUG over GUG. Among the nine species, the *H. influenzae* genome is the most AT-rich and is expected to have a high AUG% because both mutation and selection favor AUG over GUG. In contrast, GC-rich genomes have GC-biased mutations, which will favor GUG over AUG. Because the four LGB all have a higher GC% than the five species with relatively short generations, they may use fewer AUG and more GUG codons as start codons simply because of their genomic GC-richness. For this reason, it is necessary to include genomic GC as a control variable. Also, HEGs and REST genes may differ in the relationship between AUG% and the generation time. A conceptually more comprehensive and explicit model is, therefore, needed.

Here, the dependent variable is AUG% and independent variables include ranked generation time (RankGT), genomic GC content (GC%, which is taken as a proxy for a genomic mutation shared by both HEGs and REST genes), and gene expression (GE with two categories, HEGs and REST genes, encoded as 0 and 1, respectively). The input data (Table 4) are used to fit the model.

A regression analysis of the input data in Table 4 showed that all three independent variables were statistically significant, but their interaction terms were not (Table 5). The regression model was then fitted without interaction terms. This reduced model accounted for 82.0% of the variation in the dependent variable AUG%. The two regression equations derived from the regression coefficients in Table 5, one for HEGs and the other for the REST, are

(3) For HEGs: AUG%=125.86−2.54RankGT−0.52GC%

(4) For REST: AUG%=116.68−2.54RankGT−0.52GC%

Equations (3) and (4) and Table 5 show that (1) the AUG% decreases highly significantly with the generation time, and (2) the AUG% is significantly higher in HEGs than in REST genes (Table 5). However, the GC% also has a significant effect on the AUG% (*p* = 0.01825, Table 5), with the AUG% decreasing with an increasing GC%. This is consistent with the interpretation of stronger selection operating on SGB than on LGB. That is, a non-AUG start codon mutating to AUG is more strongly favored by natural selection in SGB than in LGB. 

We should emphasize that Equations (3) and (4) are descriptive models. They do not explicitly prevent AUG% from taking values smaller than 0 or larger than 1. A sigmoidal function would have been more appropriate if there were enough data for parameter estimation.

### 3.3. Differential Preference for Stop Codon UAA

As reviewed previously, the stop codon UAA exhibits the smallest readthrough error rate among the three nonsense codons [57,61,62,63,64,65,66]. Consequently, HEGs favor stop codon UAA over other stop codons in multiple bacterial species [9,67,74]. This is also true for all nine bacterial species. UAA was preferred by HEGs in *E. coli*, *B. subtilis*, *M. tuberculosis* [67], and *M. abscessus* [9]. Of the five remaining species, the proportions of UAA in REST genes and HEGs were 0.653 and 0.8816, respectively, in *V. natriegens*; 0.6365 and 0.9474 in *V. cholerae*; 0.7602 and 0.9429 in *H. influenzae*; 0.0585 and 0.2222 in *M. smegmatis*; and 0.2364 and 0.2836 in *M. leprae*. Thus, UAA is consistently preferred in HEGs relative to REST genes.

Given that UAA is the best stop codon [128,129], we predicted that SGB should use more UAA codons as stop codons than LGB. Similar to our analysis of the start codon AUG usage, the dependent variable now was UAA% and the independent variables included the ranked generation time (RankGT), genomic GC content (GC%), and gene expression (GE with two categories, HEGs and REST genes, encoded as 0 and 1, respectively). The genomic GC% is particularly relevant in studying UAA usage because protein-coding genes in a GC-rich genome tend to use more UGA and UAG stop codons than those in an AT-rich genome [67]. 

A regression analysis showed that all three independent variables were statistically significant (Table 6). The model accounted for 94.7% of the total variation in UAA%. The two regression equations derived from the regression coefficients in Table 6, one for HEGs and the other for the REST, are

(5) For HEGs: UAA%=197.95−3.78RankGT−2.22GC%

(6) For REST: UAA%=180.60−3.78RankGT−2.22GC%

Equations (5) and (6) and Table 6 show that the UAA% decreases highly significantly with an increasing generation time, and that HEGs use UAA significantly more frequently than REST genes (Table 6, a 17.35326% difference between the two). This is consistent with the interpretation of stronger selection operating on SGB than on LGB. A non-UAA stop codon mutating to UAA is more strongly favored by natural selection in SGB than in LGB. 

UAA usage also decreases significantly with an increasing genomic GC% (Table 6), which is understandable. As the genomic GC% increases, GC-biased mutations will favor UAG and UGA codons over UAA codons [67]. It is for this reason that the genomic GC content needs to be taken into consideration when assessing codon usage bias.

### 3.4. Impact of Differential Selection on Sense Codons

It is difficult to evaluate the impact of tRNA-mediated selection on codon usage across species because the codon adaptation index (CAI) [50,51] and the index of translation efficiency (*I_TE_*) [7] are both for comparing genes within species. The effective number of codons (ENC) [52,130] could potentially be used for among-species comparisons, but all these indices are strongly affected by genomic mutation bias [131,132]. For example, protein-coding genes in strongly GC-biased genomes will have mostly G-ending and C-ending codons, leading to a reduced ENC that has little to do with selection.

It is reasonable to assume that mutation bias affects both HEGs and REST genes. Thus, the difference in mean *I_TE_* between HEGs and REST genes, i.e.,

(7) $$D_{I_{TE}}=\left({\bar{I}}_{TE.HEG}-{\bar{I}}_{TE.REST}\right),$$

should be relatively independent of mutation bias. $D_{I_{TE}}$ should increase with selection intensity for translation elongation efficiency. If there is no selection for translation elongation efficiency, then $D_{I_{TE}}$ is expected to be 0. With an increasingly strong selection for translation elongation efficiency, $D_{I_{TE}}$ should also increase because the selection is expected to be stronger for HEGs than for REST genes. In other words, strong selection for translation efficiency in SGB should drive HEGs towards better codon adaptation than the REST genes, thereby increasing $D_{I_{TE}}$. 

**Figure 2:**
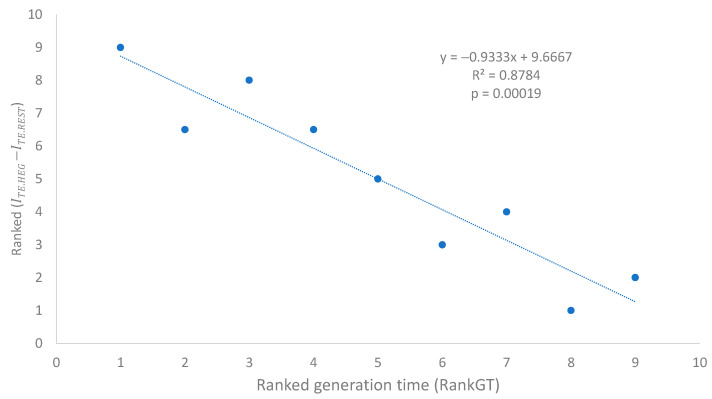
Selection for codon optimization, measured by ranked $D_{I_{TE}}\;$($={\bar{I}}_{TE.HEG}-{\bar{I}}_{TE.REST})$ in Equation (7), decreases with increasing generation time in nine bacterial species.

The $D_{I_{TE}}$ values and its ranks (Rank $D_{I_{TE}}$, Table 7) depend strongly on generation time (RankGT, Table 7). The relationship is best illustrated with two ranked variables, i.e., ranked generation time (RandGT) and ranked $D_{I_{TE}}$ (Figure 2). The fitted regression line accounts for 87.84% of the total variation in ranked $D_{I_{TE}}$ (Figure 2).

### 3.5. Differential Selection Drives tRNA Adaptation

Codons and tRNAs are expected to coevolve and adapt to each other [2,6,30,31], especially in highly expressed genes [6,7,30,31,32,33,34,35,36,133]. Given the better codon optimization in SGB than in LGB (Figure 2 and Table 7), one would predict more tRNA genes for highly used codons than rarely used codons. If we focus on the anticodons of tRNA genes, then the prediction above implies a smaller effective number of anticodons (N_AC_), equivalent to the concept of the effective number of codons [52,130], in SGB than in LGB. Specifically, N_AC_ should increase with RankGT (ranked generation time).

**Figure 3:**
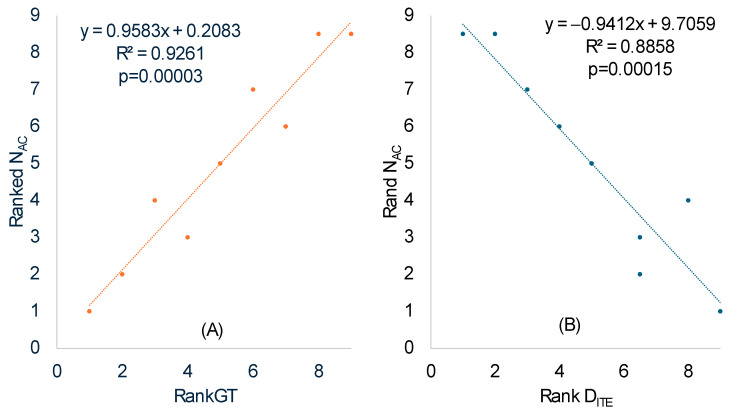
The ranked effective number of anticodons (Rank N_AC_) increases with ranked generation time (RankGT) in (**A**), and decreases with increasing codon adaptation (Rank $D_{I_{TE}}$) in (**B**).

We calculated N_AC_ in the same way the effective number of codons is calculated [52], except that codons in coding sequences were replaced by anticodons in tRNA genes. The ranked N_AC_ increased highly significantly with ranked RankGT (Table 8 and Figure 3A), consistent with our prediction that SGB should have a smaller N_AC_ than LGB. One should note the difference between a codon replacement and an anticodon replacement. A codon replacement may have only a minor effect on the translation of a single gene, but an anticodon replacement will affect the translation of numerous codons. For this reason, anticodons are strongly constrained and much less affected by genomic GC%.

We have previously used Rank $D_{I_{TE}}$ (Table 7) as a species-level measure of codon adaptation. This $D_{I_{TE}}$ is expected to be negatively associated with the ranked N_AC_ for the following reason. Better codon adaptation (high Rank $D_{I_{TE}}$) implies higher usage of major codons, which requires more tRNAs with the corresponding decoding anticodon to translate these overused major codons, leading to a decreased N_AC_ in species with a high degree of codon adaptation. This expected relationship between N_AC_ and $D_{I_{TE}}$ was substantiated empirically (Figure 3B, *p* = 0.00015).

The analysis above assumes that tRNA gene copy numbers in bacterial genomes are proportional to the abundances of tRNA molecules in the cell. With the availability of transcriptomic data, it has been found that the assumption is generally true, i.e., the copy number of a tRNA is highly correlated with the transcriptomic representation of the tRNA [134].

### 3.6. Secondary Structural Stability near the Start and Stop Codons

Because the 30S ribosomal subunit requires a single-stranded mRNA region for binding [28,29], sequences immediately flanking translation signals in bacteria (e.g., Shine–Dalgarno sequence and start and stop codons) are expected to have reduced secondary structures, to avoid embedding translation signals in a stable secondary structure [18,27,116,126,135,136]. This pattern has also been observed in bacteriophage genes [5,9]. The weakening of the secondary structure near, or immediately upstream of, the start codon has also been observed in highly expressed eukaryotic mRNAs [8,137], especially in mRNAs requiring internal ribosome entry for translation [138]. 

Secondary structural stability in RNA is typically measured by the minimum folding energy (MFE). An MFE equal to 0 means no secondary structure, and a stronger secondary structure corresponds to a more negative MFE value. Experiments involving engineered *E. coli* genes have shown that the translation initiation efficiency depends heavily on the MFE of the sequence upstream and including the start codon [3,7,14]. We followed the convention of previous studies [5,9] and measured the MFE with a sliding window of 40 nt along mRNA sequences, to quantify the change in secondary structural stability.

**Figure 4:**
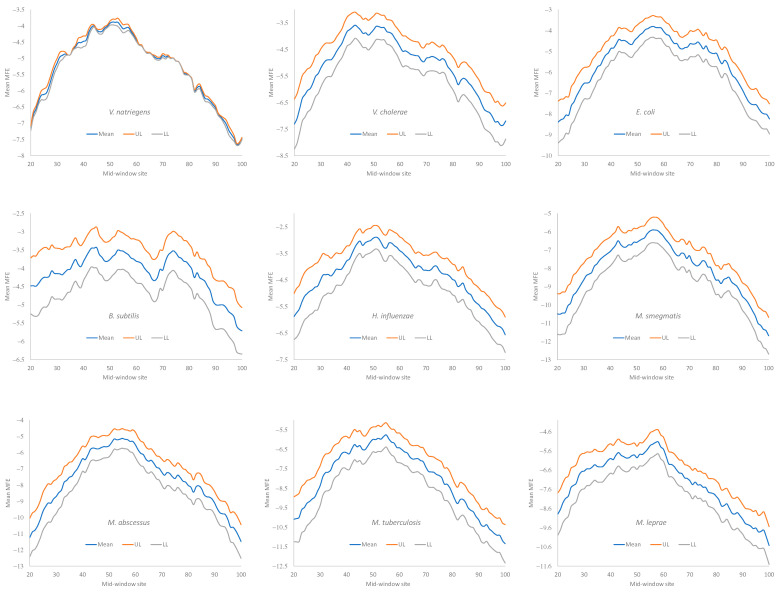
Change in MFE (minimum folding energy) over a sliding window of 40 nt in HEGs of the nine bacterial species (one sub-figure for each species). The start codon occupies sites 61–63. The mid-window site (horizontal axis) indicates the middle of the sliding window of 40 nt. The middle blue curve is the mean MFE of all HEGs (e.g., each point in the mean curve for *V. nitriegens* is the average of 76 HEGs). The two curves above and below the mean curve are the 95% upper and lower limits (UL and LL).

Secondary structural stability, as measured by MFE, decreased near the start codon, but the weakest secondary structure was observed slightly upstream of the start codon (Figure 4), corresponding to the SD sequence. This pattern has been observed before in bacteriophage genes and their host genes [5,9] and is consistent with the interpretation that a strong secondary structure embedding the SD sequence or the start codon is selected against because it prevents the translation initiation signal (SD and start codon) from being decoded by the aSD sequence and the initiation tRNA, respectively.

One might argue against the interpretation that the reduced secondary structure near the translation initiation serves to avoid embedding crucial translation initiation signals such as SD sequences and start codons in a stable secondary structure. The SD sequences are purine-rich and cannot form a secondary structure within them. Thus, the reduced secondary structure near the SD sequence (Figure 4) could be a direct consequence of the purine-richness in the SD sequences, with nothing to do with the hypothesized avoidance of the secondary structure embedding important translation initiation signals. We thus have two hypotheses. Hypothesis 1 invokes selection against secondary structural stability near the translation initiation signals. Hypothesis 2 states that the weakening of the secondary structure near the translation initiation signal is a simple consequence of the purine-rich SD, with no selection specifically against the secondary structure.

**Figure 5:**
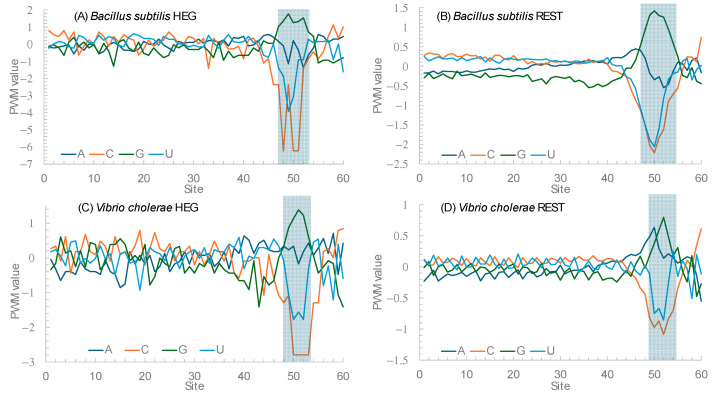
Dramatic reduction in nucleotide C near the Shine–Dalgarno sequence (shaded). Plotted are position weight matrix (PWM) scores in sequences immediately upstream of the start codon (at sites 61–63) in HEGs (**A**) and REST genes (**B**) in *Bacillus subtilis*, and in HEGS (**C**) and REST genes (**D**) in *Vibrio cholerae*.

One way to differentiate these two hypotheses is to consider the observation that SD sequences are mainly G-rich. Because G will base-pair with C, Hypothesis 1 (invoking selection against secondary structural stability) will predict a stronger avoidance of nucleotide C relative to nucleotide U. In contrast, Hypothesis 2 gives no reason to expect an avoidance of nucleotide C relative to U near the translation initiation signals. Figure 5 plots the position weight matrix (PWM) scores for the 60 nucleotides immediately upstream of the start codon for two species, *Bacillus subtilis* and *Vibrio cholarae.* PWM scores measure nucleotide usage bias relative to the background nucleotide frequencies. A value of 0 means unbiased usage, a positive value means overuse, and a negative value means avoidance. Figure 5A,B contrast the HEGs and the REST genes in *B. subtilis.* If Hypothesis 1 is correct, then we expect stronger avoidance of C relative to U in HEGs than in the REST genes. The nucleotide usage patterns in Figure 5A,B are consistent with the prediction from Hypothesis 1. An approximate statistical test can be performed as follows. Within sites 45 to 55, there 36 nucleotide Cs and 129 nucleotide Us in the HEGs. The corresponding numbers are 2952 C and 7069 U. The percent of C is 21.818% in the former and 29.458% in the latter. The two are significantly different (likelihood ratio chi-square = 4.84, DF = 1, *p* = 0.0278). If we narrow the range to sites 48 to 53, then the difference becomes more significant. The nucleotide usage patterns for HEGs and REST genes in *V. cholerae* are similar (Figure 5C,D).

All nine bacterial species exhibited a decreased secondary structure near the translation initiation signals (Figure 4). However, we did not take into account the effect of the GC%. Increasing the GC% is expected to increase secondary structural stability in mRNA. As we can see from Table 9, *H. influenzae* has the lowest genomic GC% (38.2%), and its mean MFE is the closest to 0 (which means no secondary structure). In contrast, *M. smegmatis* and *M. tuberculosis* have the highest genomic GC%, and their mRNAs tend to have more negative MFE values (Figure 4). Accordingly, without controlling for GC%, the observation that the LGB have a stronger secondary structure than the SGB cannot be attributed to a reduced selection in these LGB against a stable secondary structure. Note that the MFE and GC% were calculated for each sequence, and their averages are presented in Table 9 as the MeanMFE and GC%, respectively.

Given that a weak secondary structure near the SD sequence and the start codon is favorable (Figure 4), one would predict that SGB should have weaker secondary structures (larger MFE values) than LGB. In order to test this prediction while accommodating the effect of the GC%, we characterized the MFE plots of sliding windows for each species using a single value for each. That is, for each plot in Figure 4, we calculated the mean value for mid-window sites 46–65 (with the start codon occupying sites 58–60). These sites included both the SD sequence and the start codon. The mean MFE (Table 9) was now the dependent variable. It was expected to (1) decrease (i.e., more stable secondary structure) with an increasing generation time (RankGT) and increasing GC%, and (2) increase with gene expression (GE, i.e., be greater for HEGs than for REST genes). These three independent variables are also included in Table 9.

The best model, which accounts for 98.2% of the total variation in MeanMFE in Table 9, included the three dependent variables and an interaction term (Table 10). The two-tailed p for RankGT was 0.064 (Table 10). However, because we had an explicit one-tailed prediction of a negative slope (i.e., MeanMFE should decrease with increasing RankGT), p was half of 0.064, i.e., 0.032. Other regression terms for GC%, GE, and their interaction were highly significant and consistent with the predictions (Table 10).

As before, we give the two regression equations separately for HEGs and the REST from the regression coefficients in Table 10:

(8) For HEGs:MeanMFE=0.57408−0.08382 RankGT−0.10038 GC%

(9) For REST:MeanMFE=5.03448−0.08382 RankGT−0.21009 GC%

Equations (8) and (9) show that, for both HEGs and REST genes, secondary structural stability increases with the generation time (MFE becomes more negative with an increasing generation time). This is consistent with our prediction that selection against the secondary structure near the translation start signals (SD sequence and start codon) is stronger in SGB than LGB.

MeanMFE decreases more sharply with the GC% in Equation (9) than in Equation (8), i.e., secondary structural stability increases more rapidly with the GC% for REST genes than for HEGs. This is easy to understand if the selection against secondary structural stability is on average stronger in HEGs than in REST genes. The MeanMFE decreases by only 0.10038 (Equation (8)) for a unit increase in the GC% with the strong selection of HEGs, but decreases by 0.21009 (Equation (9)) for the same unit change in the GC% with the relatively weak selection of REST genes.

**Figure 6:**
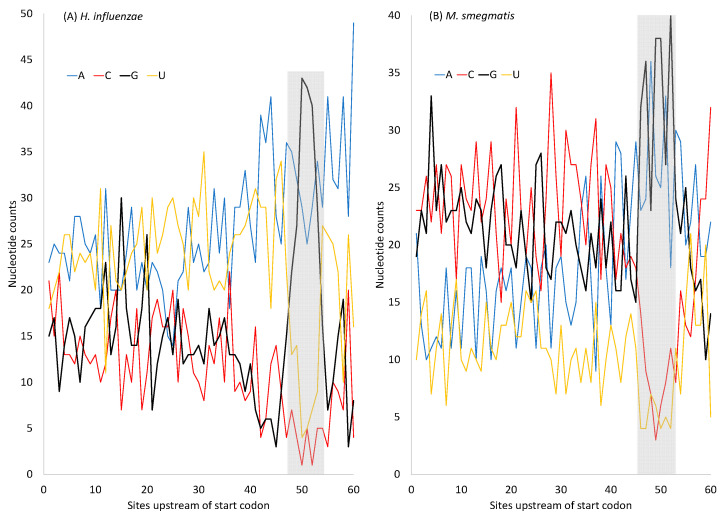
Changes in nucleotide frequencies in sequences immediately upstream of the start codon (at sites 61–63) in highly expressed genes (HEGs). Note the sharp increase in nucleotide G and concurrent decrease in nucleotides C and U near site 50 corresponding to the Shine–Dalgarno (SD) sequence (shaded). (**A**) AT-rich *H. influenzae.* (**B**) GC-rich *M. smegmatis*.

The selectionist interpretation above does not consider the effect of mutations, which offers an alternative interpretation. In general, spontaneous mutations in AT-rich genomes tend to be AT-biased, based on (1) comparisons between pseudogenes and their functional counterparts [139,140], (2) the mutation patterns of pathogenic bacteria with relaxed selection [141,142], and (3) nucleotide bias at the three codon sites across multiple bacterial species [143]. *H. influenzae* has an AT-rich genome, suggesting AT-biased mutation, in contrast to *M. smegmatis,* which has a GC-rich genome. However, protein-coding genes in both species have SD sequences that are purine-rich (especially G-rich) (Figure 6). The G-rich SD will form base-pairs with nearby C nucleotides, so the MFE will not fall to 0. In pseudogenes where selection for maintaining the G-rich SD sequence is absent, or in low-expressed genes where the selection is weak, the AT-rich *H. influenzae* will lose these G nucleotides in the SD sequence, leading to an MFE closer to 0. Indeed, the MeanMFE value for the 19 pseudogenes in *H. influenzae* is −2.2887, closer to 0 than all MeanMFE values in Table 9. Similarly, the MeanMFE is −3.2660 for HEGs and −2.5466 for REST genes (Table 9). This is consistent with the interpretation that the G-rich SD is more likely to be hit by G→A and G→T mutations and lose G/C base-pairs in REST genes than in HEGs. For example, *H. influenzae* has a GC% of 37.9329% for HEGs but only 33.7986% for REST genes, leading to a MeanMFE value closer to 0 in REST genes than in HEGs. 

In short, although the purine-rich SD sequences (Figure 6) can hardly form secondary structures within themselves, the dramatically increased G nucleotides within the SD sequence could base-pair with the neighboring C nucleotides and contribute to secondary structural stability. If there is no selection maintaining the G-richness in the SD sequences, then these G nucleotides may be replaced by A and T, leading to a further decrease in secondary structural stability. Thus, both selection and mutation could contribute to secondary structural stability in sequences near the translation initiation signals (the SD sequence and the start codon). The models in Equations (8) and (9) are, therefore, oversimplified and should be interpreted with caution. The secondary structure in sequences near the stop codon exhibits a similar pattern to those near the start codon (Figure 6).

The decrease in secondary structural stability may not necessarily be related to the avoidance of embedding SD sequences and start codons. Efficiently translated yeast mRNAs (i.e., mRNAs in polysomes with high ribosome densities) often have a short poly(A) tract before the start codon [137], with the poly(A) interpreted as long enough to recruit translation initiation factors but short enough to avoid binding by the poly(A)-binding proteins. However, the presence of poly(A) also weakens the secondary structural stability as a secondary consequence.

## 4. Discussion

**Figure 7:**
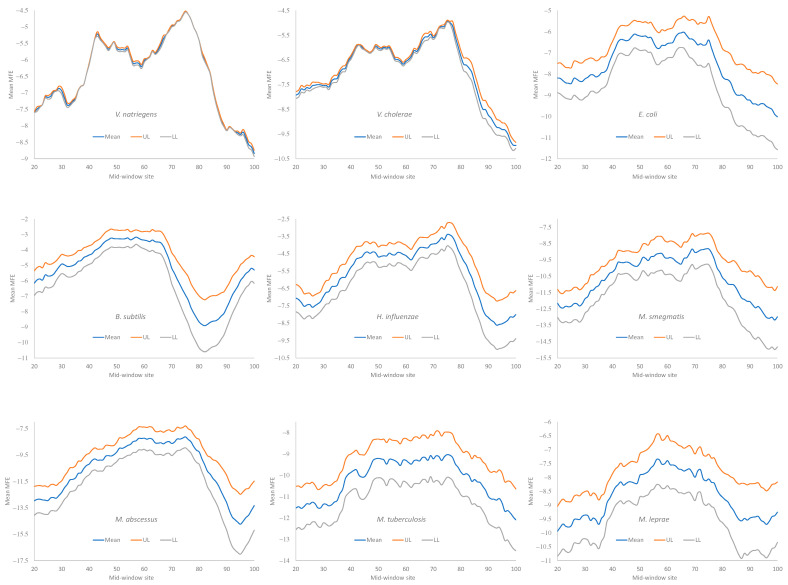
Change in MFE (minimum folding energy) over a sliding window of 40 nt in HEGs of the nine bacterial species. The stop codon occupies sites 58–60. Other annotations are identical to those in Figure 4.

Our assumption that SGB are under stronger selection for translation efficiency than LGB appears to be valid because multiple predictions based on the assumption are consistent with the empirical evidence. First, the number of ribosome RNA operons, as well as the number of tRNA genes, increases with a decreasing generation time (Table 2 and Figure 1). Second, AUG is known to be the most efficient start codon, and SGB genes exhibit a stronger preference for AUG as a start codon than LGB, especially in highly expressed genes (HEGs) (Table 3, Table 4 and Table 5). This is also true in the usage of stop codon UAA (Table 6), which is known to be the most efficient termination signal with the smallest readthrough error rate as a stop codon. Third, SGB, especially their HEGs, exhibits better codon and anticodon adaptation than LGB (Table 7 and Figure 2 for codons, and Table 8 and Figure 3). Finally, SGB genes have weaker secondary structures near translation initiation signals than LGB (Figure 4 and Table 9 and Table 10). A similar pattern was observed with sequence secondary structures near the stop codon (Figure 7). However, as we discussed previously, the selectionist interpretation is sometimes confounded by biased mutations.

One may ask why there should ever be LGB with weak selection for translation optimization, given natural selection operating to maximize growth and reproduction. There are different environments in which translation efficiency may not be a limiting factor for growth and reproduction. For example, *M. tuberculosis* wraps itself with a thick layer of mycolic acids that serves two functions [144]: (1) to prevent antibiotics from reaching the cell membrane [145], and (2) to evade the attack of the host immune system [146]. However, after being phagocytosed by pulmonary macrophages and confined within the phagosome and the granuloma, *M. tuberculosis* survives in an extremely nutrient-limited environment and adopts a prolonged stage of dormancy with no chance of rapid growth [109]. Mutations still occur during this latent stage of dormancy [147,148] in both protein-coding genes [149] and rRNA genes [150,151]. Thus, mutations without the checking of natural selection will lead to a suboptimal translation machinery in *M. tuberculosis.* Such a suboptimal machinery cannot perform efficient translation even when the bacteria are not nutrient-limited. This is in dramatic contrast to *E. coli,* which experiences rapid alternation of feast and famine cycles several times each day, with natural selection eliminating those mutants that cannot translate efficiently during the feast period. 

Modern biological research aims to formulate and validate quantitative and mechanistic models. In this context, this study has several shortcomings. The first shortcoming is the inherent inaccuracy of generation time. The experimentally measured generation time sometimes varies widely among strains and among different studies. For example, the generation time is ~21 h in drug-sensitive strains of *M. tuberculosis*, but ~35 h in the multi-drug-resistant strains [152]. This suggests a cost to the pathogen in developing multi-drug resistance, i.e., the resistance is at the cost of longer generations. However, the observation also highlights the inherent variation in measured generation time. Particularly controversial is what generation time to use. For example, the generation time in *E. coli* is only about 20–30 min under favorable culture conditions but could be substantially longer in the natural habitat of the mammalian intestine, estimated by the rate of mutation accumulation [153]. To model the joint effect of mutation and selection, the generation time under natural conditions would seem more appropriate than that under optimal experimental conditions. Unfortunately, the generation time in bacterial populations in nature cannot be measured accurately. For example, the estimated generation of 15 h for *E. coli* [153] is associated with a 95% confidence interval of 0–30 h. Another uncertainty with generation time is that we do not know if long-generation species such as *M. leprae* really cannot replicate fast or if they could grow fast but microbiologists have not been able to shift them into the fact-growing mode. Because of the uncertainty in generation times among the bacterial species, we ranked the generation times in the hope that, relative to one another, they would be in the correct order. It is for this reason that we chose species with widely different generation times characterized experimentally, so that our ranking of the generation times would not be controversial.

The second shortcoming of this study is the small number of bacterial species, partly because of our conscious effort to avoid species with uncertain generation times. There are various tables of bacterial generation times for more than nine bacterial species, but they often do not include original references or do not have fully sequenced genomes. Some bacterial species in Mollicutes have known generation times, e.g., ~6 h in *Mycoplasma pneumoniae* [154,155], as well as fully sequenced genomes. However, their genetic code (i.e., translation table 4) differs from the rest of the bacteria (translation table 11). This complicates comparisons. Consequently, they are not included in this study. The small number of bacterial species included here results in two limitations. First, it does not permit the validation of parameter-rich models. Second, it does not allow for phylogeny-based inference [156,157,158] to alleviate the issue of data dependence. We have previously used such phylogeny-based inference to quantify the relationships between body temperature and genome size [159] and between the optimal growth temperature and ribosomal RNAs’ secondary structural stability (i.e., the stem length and GC% in the stem–loop structure in rRNAs) [98]. The phylogenetically independent contrasts showed that the stem–loop structure in bacterial thermophiles tended to have longer and more GC-rich stems than that in mesophiles [98] and that poikilotherms in warm climates tended to have smaller genomes than those in cold climates [159]. In this context, we may highlight two points. First, our results are highly consistent with the prediction that increasing the generation time decreases the intensity of selection on translation efficiency. Second, the bacterial species we use are highly divergent. Even the two most closely related species, *M. tuberculosis* and *M. leprae*, have an evolutionary distance of more than 0.3 even for conserved ribosomal protein genes [160], so phylogenetic inertia might not affect the quantified relationships, i.e., the data points might be considered to be roughly independent.

The third shortcoming is the lack of a general conceptual framework for the impact of mutation and selection on translation optimization, one that can be used across species. Protein production from an mRNA depends on the ribosomal recruitment rate, the efficiency in forming the 70S initiation complex, the elongation efficiency and accuracy, the termination efficiency and accuracy, the stability of the mRNA, the differential amino acid and tRNA availability, and the energy level of the cell [4,18,161,162]. How do these variables interact with each other to affect protein production? For example, when translation is not efficient, codon usage optimization has little effect on protein production. However, protein production increases significantly with codon optimization in mRNAs with efficient initiation [3,7,14]. Similarly, there are conflicts between maximizing transcription efficiency and translation efficiency. An RNA will be transcribed efficiently if it maximizes the usage of the abundant nucleotide A and minimizes the rare nucleotide C [163,164]. However, this will drive up the usage of A-ending codons that may not be the optimal codons for translation. There are also factors affecting codon usage that are not related to translation. For example, mammalian species have zinc-finger antiviral proteins (ZAPs), which work against RNA viruses by targeting CpG dinucleotides in the viral RNA [165,166,167,168,169]. Many human RNA viruses exhibit much reduced CpG dinucleotides [165,170,171,172,173], with SARS-CoV-2 being the extreme among coronaviruses [174,175]. Most of the CpG reduction occurs at the di-codon configuration, from NNC GNN to NNT GNN (i.e., a synonymous replacement). This NNC to NNU change has nothing to do with codon optimization. So, what will be the functional relationship among all these variables? The model is even more complicated if we consider not only HEGs but also genes whose optimal protein level is not the maximal, such as the autoregulated level of release factor 2 in *E. coli* [82,83] or many others [176]. We highlight such questions in the hope that they will motivate researchers to address them.

The last point we wish to discuss is the usage of ranked variables such as the ranked generation time (RankGT). Specifically, the difference in generation time (GT) between the fast-growing *Vibrio natriegens* and *V. cholerae* is only a few minutes, whereas that between the slow-growing *Mycobacterium tuberculosis* and *M. leprae* is about six days. After completing a ranking, the difference in RankGT between the two fast-growing species becomes one (=2–1), which is the same as the difference in RankGT between the two slow-growing species (=9–8). This implicitly assumes that the difference of a few minutes in GT between the two fast-growing species is roughly equivalent to the differences of about six days in GT between the two slow-growing species. 

Our results seem to substantiate the assumption above. For example, *Mycobacterium tuberculosis* and *M. leprae* have one *rrn* operon in their genomes. Any species with a much longer generation time in months or even years will not reduce the number of *rrn* operons below one. Thus, adding many days to the generation time in a slow-growing species will not change the number of *rrn* operons, but shortening the generation time by a few minutes in a fast-growing species may well increase the number of *rrn* operons. This suggests that a difference of a few minutes in GT in fast-growing species may actually result in a greater effect than a difference of a few days in slow-growing species. The same applies to the number of tRNA genes (Figure 1) and might be applicable to other translation-related features. 

## 5. Conclusions

Detection of the impact of selection on translation efficiency is mostly performed by contrasting highly expressed genes and low-expressed genes within rapidly replicating species. We generalized such investigations to determine how differential selection for translation efficiency among different species will leave its footprints on the species-specific translation machinery. Our results suggest that selection for translation optimization is stronger in short-generation species than long-generation species, and that this differential selection strongly shapes the evolutionary trajectories of translation machineries in these species, affecting translation initiation, elongation, and termination.

## Figures and Tables

**Table 1:** Nine species with complete genomes and well-documented generation times (GTs) under optimal growth conditions.

Species	Accession ^(1)^	OGT ^(2)^	GT ^(3)^	Rank ^(4)^	Ref. ^(5)^
*Vibrio natriegens*	NZ_CP009977, NZ_CP009978.1	37 °C	10 min.	1	[100,101]
*Vibrio cholerae*	NZ_CP043554, NZ_CP043556.1	37 °C	16–20 min.	2	[102]
*Escherichia coli*	NC_000913.3	37 °C	20–30 min.	3	[103]
*Bacillus subtilis*	NC_000964.3	37 °C	30–70 min.	4	[104]
*Haemophilus influenzae*	NZ_CP007470.1	37 °C	103–107 min.	5	[105]
*Mycolicibacterium smegmatis*	NZ_CP054795.1	37 °C	~2 h	6	[106]
*Mycobacterioides abscessus*	NZ_CP034181.1	36 °C	4–5 h	7	[107]
*Mycobacterium tuberculosis*	NC_000962.3	37 °C	20–30 h	8	[15,108,109]
*M. leprae*	NZ_CP029543.1	30 °C	7 days	9	[15,110]

^(1)^ GenBank accession number. ^(2)^ Optimal growth temperature. ^(3)^ Generation time under optimal growth conditions. ^(4)^ Ranking of generation time from smallest to largest. ^(5)^ References pertaining to the generation time.

**Table 2:** Short generation times are associated with increased genomic investment in ribosomal RNA and tRNA.

Species	GT ^(1)^	L_Genome_^(2)^	N_CDS_^(3)^	N_rrn_^(4)^	N_tRNA_^(5)^
*Vibrio natriegens*	1	5,175,153	4496	11	129
*Vibrio cholerae*	2	4,089,299	3628	10	102
*Escherichia coli*	3	4,641,652	4298	7	86
*Bacillus subtilis*	4	4,215,606	4237	10	86
*Haemophilus influenzae*	5	1,846,259	1714	6	59
*Mycolicibacterium smegmatis*	6	6,993,871	6540	3	47
*Mycobacterioides abscessus*	7	5,067,231	4938	1	47
*Mycobacterium tuberculosis*	8	4,411,532	3905	1	45
*Mycobacterium leprae*	9	3,187,112	2328	1	45

^(1)^ Ranking of generation times from the shorted to the longest. ^(2)^ Genome length. The two Vibrio species each have two chromosomes. The genome length is the sum of the two chromosomes. ^(3)^ Number of protein-coding genes. ^(4)^ Number of *rrn* operons in the genome. ^(5)^ Number of tRNA genes in the genome.

**Table 3:** Start codon usage and percentage of AUG (AUG%) in highly expressed protein-coding genes (HEGs) and the rest of the protein-coding genes (REST).

Species	HEGs					REST					
	AUG	GUG	YUG ^(1)^	N_HEG_^(2)^	AUG%	AUG	GUG	YUG ^(1)^	AUH ^(1)^	N_REST_^(2)^	AUG%
*V. natriegens*	71	3	2	76	93.42	3976	297	124	15	4412	90.12
*V. cholerae*	70	3	3	76	92.11	3166	253	131	18	3568	88.73
*E. coli*	69	3	1	73	94.52	3805	335	81	4	4225	90.06
*B. subtilis*	58	5	3	66	87.88	3225	382	555	9	4171	77.32
*H. influenzae*	68	1	1	70	97.14	1554	51	28	10	1643	94.58
*M. smegmatis*	54	18	0	72	75.00	4255	1998	187	26	6466	65.81
*M. abscessus*	53	14	1	68	77.94	3255	1458	145	14	4872	66.81
*M. tuberculosis*	49	13	0	62	79.03	2357	1306	177	4	3844	61.32
*M. leprae*	48	16	3	67	71.64	1162	782	276	43	2263	51.35

^(1)^ YUG includes CUG and UUG; AUH includes AUA, AUC, and AUU. ^(2)^ N_HEG_: the number of highly expressed genes; H_REST_: the number of all other genes not in the HEG group.

**Table 4:** The percentage of AUG codons (AUG%) acting as start codons depends on the generation time (RankGT), genomic GC content (GC%), and gene expression (GE).

Species	AUG%	RankGT	GC%	GE
*V. natriegens*	93.4211	1	45.0	HEG
*V. cholerae*	92.1053	2	47.3	HEG
*E. coli*	94.5205	3	50.8	HEG
*B. subtilis*	87.8788	4	43.5	HEG
*H. influenzae*	97.1429	5	38.2	HEG
*M. smegmatis*	75.0000	6	67.4	HEG
*M. abscessus*	77.9412	7	64.1	HEG
*M. tuberculosis*	79.0323	8	65.6	HEG
*M. leprae*	71.6418	9	57.8	HEG
*V. natriegens*	90.1179	1	45.0	REST
*V. cholerae*	88.7332	2	47.3	REST
*E. coli*	90.0592	3	50.8	REST
*B. subtilis*	77.3196	4	43.5	REST
*H. influenzae*	94.5831	5	38.2	REST
*M. smegmatis*	65.8058	6	67.4	REST
*M. abscessus*	66.8103	7	64.1	REST
*M. tuberculosis*	61.3163	8	65.6	REST
*M. leprae*	51.3478	9	57.8	REST

**Table 5:** Regression analysis of data in Table 4, with AUG% as the dependent variable, and ranked generation time (RankGT), genomic GC content (GC%), and gene expression (with HEG and REST encoded as 0 and 1, respectively) as the independent variables.

	Coefficient	Standard Error	t Stat	*p*-Value
Intercept	125.85528	8.60272	14.62970	0.00000
RankGT	−2.53863	0.76412	−3.32229	0.00503
GC%	−0.52069	0.19491	−2.67147	0.01825
GE	−9.17674	2.94435	−3.11673	0.00758

**Table 6:** Regression analysis of the impact of UAA% on three independent variables: ranked generation time (RankGT), genomic GC content (GC%), and gene expression (with HEG and REST encoded as 0 and 1, respectively).

	Coefficient	Standard Error	t Stat	*p*-Value
Intercept	197.94875	11.48080	17.24172	0.00000
RankGT	−3.77935	1.01976	−3.70611	0.00235
GC%	−2.21876	0.26012	−8.52992	0.00000
GE	−17.35326	3.92940	−4.41627	0.00059

**Table 7:** Differences in mean *I_TE_* values between HEGs and REST genes ($D_{I_{TE}}$) for the nine bacterial species at different generation times (RankGT).

Species	RankGT	$D_{I_{TE}}$	$\mathrm{Rank}\;D_{I_{TE}}$
*V. natriegens*	1	0.2516	9
*V. cholerae*	2	0.2380	6.5
*E. coli*	3	0.2465	8
*B. subtilis*	4	0.2380	6.5
*H. influenzae*	5	0.2092	5
*M. smegmatis*	6	0.1266	3
*M. abscessus*	7	0.1806	4
*M. tuberculosis*	8	0.0267	1
*M. leprae*	9	0.0689	2

**Table 8:** The effective number of anticodons (N_AC_ and its ranked N_AC_) increases with ranked generation time (RankGT).

Species	RankGT	N_AC_	Rank N_AC_
*V. natriegens*	1	40.2129	1
*V. cholerae*	2	44.2802	2
*E. coli*	3	49.0127	4
*B. subtilis*	4	45.8531	3
*H. influenzae*	5	50.4305	5
*M. smegmatis*	6	59.0667	7
*M. abscessus*	7	58.8153	6
*M. tuberculosis*	8	59.3744	8.5
*M. leprae*	9	59.3744	8.5

**Table 9:** Secondary structural stability (measured by MeanMFE) is expected to change with generation time (RankGT), GC%, and gene expression (GE).

Species	RankGT	GC%	GE	MeanMFE
*V. natriegens*	1	42.8557	HEG	−4.2351
*V. cholerae*	2	43.7813	HEG	−4.0721
*E. coli*	3	46.5955	HEG	−4.2153
*B. subtilis*	4	38.7556	HEG	−3.7484
*H. influenzae*	5	37.9329	HEG	−3.2660
*M. smegmatis*	6	60.4224	HEG	−6.4462
*M. abscessus*	7	60.4048	HEG	−5.5966
*M. tuberculosis*	8	61.5043	HEG	−6.2934
*M. leprae*	9	56.5344	HEG	−5.7794
*V. natriegens*	1	40.4234	REST	−3.5238
*V. cholerae*	2	43.3125	REST	−4.0330
*E. coli*	3	45.6418	REST	−4.4059
*B. subtilis*	4	38.6014	REST	−3.7406
*H. influenzae*	5	33.7986	REST	−2.5466
*M. smegmatis*	6	63.6954	REST	−8.6708
*M. abscessus*	7	61.2667	REST	−8.3538
*M. tuberculosis*	8	63.0856	REST	−9.1141
*M. leprae*	9	57.1858	REST	−7.9865

**Table 10:** Regression output based on data in Table 9, with GE encoded as a binary dummy variation (0 for HEG and 1 for REST). “GC%* GE” is the interaction term.

	Coefficients	Standard Error	t Stat	*p*-Value
Intercept	0.57408	0.61265	0.93704	0.36582
RankGT	−0.08382	0.04142	−2.02386	0.06404
GC%	−0.10038	0.01414	−7.09756	0.00001
GE	4.46040	0.75315	5.92236	0.00005
GC%*GE	−0.10972	0.01483	−7.39673	0.00001

## Data Availability

Data are contained within the article and Supplementary Materials.

## Supplementary Materials

The following supporting information can be downloaded at: https://www.mdpi.com/article/10.3390/microorganisms12040768/s1, Table S1: Highly expressed genes and their locus tags in the nine bacterial species.

## Abbreviations

AC: tRNA anticodon; aSD: anti-Shine–Dalgarno sequence on small subunit rRNA; CAI: codon adaptation index; CDS: coding sequence; ENC: effective number of codons; GT: generation time; HEG: highly expressed genes; *I_TE_*: index of translation elongation; LEG: low-expressed genes; LGB: long-generation bacteria; MFE: minimum folding energy; OGT: optimal growth temperature; RankGT: ranked generation time; REST: protein-coding genes not in the HEG group; RF: release factor; SD: Shine–Dalgarno sequence on mRNA; SGB: short-generation bacteria.
